# Day-to-day variability in accelerometer-measured physical activity in mid-aged Australian adults

**DOI:** 10.1186/s12889-023-16734-0

**Published:** 2023-09-28

**Authors:** Ruth Brady, Wendy J. Brown, Gregore I. Mielke

**Affiliations:** 1 Department of Public Health and Sport Sciences, Faculty of Health and Life Sciences, University of Exeter, Devon, UK; 2 School of Human Movement and Nutrition Sciences, The University of Queensland, (#26B), Rm 319, St Lucia Campus, Brisbane, QLD 4072 Australia; 3 Faculty of Health Sciences and Medicine, Bond University, Gold Coast, Australia; 4 School of Public Health, The University of Queensland, Brisbane, QLD 4006 Australia

**Keywords:** Physical activity, Physical activity monitor, Devices, Accelerometer, Coefficient of variation, Variability, GGIR

## Abstract

**Purpose:**

The aim was to use accelerometer data to describe day-to-day variability in physical activity in a single week, according to sociodemographic variables, in mid-aged Australian adults.

**Methods:**

Data were from participants in the How Areas in Brisbane Influence HealTh and AcTivity (HABITAT) study who took part in a 2014 sub-study (*N* = 612; Mean age 60.6 [SD 6.9; range 48-73]). Participants wore a triaxial accelerometer (ActiGraph wGT3X-BT) on their non-dominant wrist for seven days, and data were expressed as acceleration in gravitational equivalent units (1 m*g* = 0.001 g). These were, used to estimate daily acceleration (during waking hours) and daily time spent in moderate-vigorous physical activity (MVPA, defined as ≥ 100m*g*). Coefficient of variation (calculated as [standard deviation/mean of acceleration and MVPA across the seven measurement days] * 100%) was used to describe day-to-day variability.

**Results:**

Average values for both acceleration (24.1-24.8 m*g*/day) and MVPA (75.9-79.7 mins/day) were consistent across days of the week, suggesting little day-to-day variability (at the group level). However, over seven days, average *individual* day-to-day variability in acceleration was 18.8% (SD 9.3%; range 3.4-87.7%) and in MVPA was 35.4% (SD 15.6%; range 7.3-124.6%), indicating considerable day-to-day variability in some participants. While blue collar workers had the highest average acceleration (28.6 m*g*/day) and MVPA (102.5 mins/day), their day-to-day variability was low (18.3% for acceleration and 31.9% for MVPA). In contrast, variability in acceleration was highest in men, those in professional occupations and those with high income; and variability in MVPA was higher in men than in women.

**Conclusion:**

Results show group-level estimates of average acceleration and MVPA in a single week conceal considerable day-to-day variation in how mid-age Australians accumulate their acceleration and MVPA on a daily basis. Overall, there was no clear relationship between overall volume of activity and variability. Future studies with larger sample sizes and longitudinal data are needed to build on the findings from this study and increase the generalisability of these findings to other population groups.

**Supplementary Information:**

The online version contains supplementary material available at 10.1186/s12889-023-16734-0.

## Introduction

The benefits of physical activity for individual and public health are well known. Current national and international evidence-based guidelines for physical activity encourage adults (18–64 years) to accumulate 150–300 mins of moderate-intensity physical activity, or 75–150 mins of vigorous-intensity physical activity, or an equivalent combination of moderate- and vigorous-intensity activity throughout the week for health benefits [1]. These guidelines are based on high-level evidence, mostly generated from epidemiological studies that used self-reported measures of physical activity to classify individuals based on their total weekly physical activity volume (i.e. *frequency* x *duration* x *intensity*) [2].

In recent years, increased use of accelerometers to derive proxy measures of physical activity in population-based studies has enabled researchers to examine how overall weekly volumes of physical activity are accumulated, for example in varying amounts on different days of the week. This is possible because accelerometers capture high resolution (e.g., second by second) data with day and time stamped outputs [3–6] and therefore permit analysis of the temporal sequence of activity accumulation, often referred to as the ‘*pattern*’ of physical activity.

Compared with traditional count-based approaches [5, 7, 8], analysis of raw accelerometer data allows increased control over data processing methods. The more traditional count-based approach is brand and/or model specific, and often uses proprietary algorithms [9]. In contrast, raw data facilitate transparent analyses, and enable comparisons between studies, regardless of brand or device [10]. GGIR is an open-access code which processes, analyses and converts accelerometry data into estimates of time spent in different activity intensities [11].

In a recent systematic review of studies published until March 2021, we identified 52 studies that investigated individual constructs (i.e., *intensity*, *frequency*, *duration*) of accelerometer-measured physical activity and their associations with a range of health outcomes in adults. Overall, when physical activities accumulated in different intensities and daily/weekly frequencies were compared, there were no differences in the relationships with most health outcomes. However, variations in data collection and processing methods made it difficult to compare study results, or to say with any certainty whether the effect of a given volume of activity on health outcomes was modified by the pattern in which it was accumulated. Of the 52 studies, only nine compared associations between different *frequencies* of physical activity with health outcomes; they identified variations in both daily (i.e., time of day) and weekly (i.e., days of the week) variability [12].

It is likely that individuals with different demographic (e.g., gender and age) and socioeconomic characteristics (e.g., occupation, income, and education) accumulate their weekly physical activity in diverse ways. The demands of different lifestyles almost certainly mean that people will find different ways to incorporate physical activity into their lives, in order to meet physical activity recommendations [1]. Understanding how individuals from different socioeconomic groups accumulate their physical activity may help to inform tailored physical activity interventions for those who are least active. From previous research, we understand that men, young adults, and those in more advantaged socioeconomic groups are most likely to meet weekly physical activity recommendations [13]. However, little is known about day-to-day variation in physical activity, or how individuals with different socioeconomic characteristics accumulate their weekly activity, for example by being active on only one or two days each week, or equally active on all days of the week.

The aim of this study was to use accelerometer data to describe day-to-day variability (hereafter referred to as “variability”) in physical activity in a single week according to sociodemographic variables, in mid-aged Australian adults. The objectives were to: (1) describe the overall (group) distributions (means, standard deviations, ranges) of daily acceleration (during waking hours) and minutes spent at acceleration ≥ 100m*g* (which we defined as moderate-to-vigorous physical activity, MVPA) on each day in a single week; (2) estimate the variability in acceleration and MVPA over seven days, and compare this in different sociodemographic groups; and (3) describe the variability profiles (low, mid, and high) for categories of low, moderate and high acceleration and MVPA according to gender, age, occupation, education, and household income.

## Methods

Data were from participants in the How Areas in Brisbane Influence HealTh and AcTivity (HABITAT) study who participated in a 2014 sub-study. The HABITAT study protocol has been published previously [14]. In brief, HABITAT is a longitudinal multi-level study which included adults aged 40–65 years, who were residents in Brisbane, Australia in 2007 (*N* = 11,035). HABITAT participants were surveyed by mail in 2007, 2009, 2011 and 2013. In 2014, 767 participants who responded to the previous four mail surveys were randomly selected to participate in a sub-study to collect objective measures of physical functioning and physical activity. The protocol for the sub-study has also been previously published [15–17]. HABITAT received ethical clearance from the Queensland University of Technology Human Research Ethics Committee (ID numbers: 3967H & 1300000161).

### Sociodemographic Variables

Sociodemographic variables in this study were assessed using information reported in the 2014 sub-study questionnaire, and categorised as follows: gender (men, women); age (years: 48–54, 55–59, 60–64, 65–74); occupation (professional ([professionals and managers], blue collar [technicians and trades workers and labourers and machinery operators and drivers], white collar [community and personal service workers and sales workers], office workers [clerical and administrative workers], not in the labour force [permanently unable to work, student or retired]); education (year 12 or less, diploma or certificate, bachelor degree or higher); and annual household income (AUD, < $41,599, $41,600-$72,799, $72,800-$129,999, ≥ $130,000).

### Accelerometer

Participants were asked to wear an ActiGraph wGT3X-BT (ActiGraph Corp, Pensacola, FL, USA) for seven continuous days on their non-dominant wrist. Participants were asked to remove this during sleep and water-based activities (such as showering, bathing, or swimming) and were provided with an activity log to record when and why the accelerometer was removed. The accelerometer recorded raw acceleration at a sampling frequency of 30 Hz in three axes and exported raw data expressed in gravitational equivalent units (*g*) (1 *g* = 9.81 m/s^2^).

### Accelerometer data processing

Accelerometer files (.gt3x) were downloaded using ActiLife and saved in their raw file format (.csv) to facilitate data analysis using R (R studio, 3.6.2, Boston, USA; https://www.rstudio.com/) package GGIR (version 2.0–0) [11]. Signal processing in GGIR includes the following steps 1) autocalibration using local gravity as a reference [18], 2) detection of sustained abnormally high values, 3) detection and estimation of non-wear [7] using a previously published algorithm, and 4) calculation of the average magnitude of dynamic acceleration, i.e., the vector magnitude of acceleration during all waking hours, corrected for gravity. GGIR converts the vector magnitude of acceleration into one value, referred to as Euclidean Norm Minus One (ENMO) [11], which was calculated using the following formula: ENMO (m*g*) = $\sqrt{{x}^{2}+ {y}^{2}+ {z}^{2}}-1g$ (where x, y and z axes are the accelerometer measured planes of movement). GGIR summarises the average ENMO over all the available data, normalised per 24-h cycle, with invalid data imputed by the average at similar timepoints on different days of the week.

### Physical activity variables

In this study, ENMO values, henceforth referred to as “acceleration”, were used to quantify acceleration related to the movement registered, expressed in millgravity (m*g*) units.

Time in MVPA was based on identification of five second epochs when mean acceleration was at or above 100 m*g.* This threshold, which was generated in a laboratory calibration study with 29 healthy adults aged 18–65 [10], has been widely used in population-based studies to define moderate-to-vigorous intensity physical activity [12, 15, 19, 20].

Only those participants who provided least 10 hrs of valid wear time on all seven days were included in the analytical sample (*N* = 612). Average mean accelerometer wear time across the analytical sample (*N* = 612) was 15.9 (range: 8–24) hrs/day.

### Day-to-day variability

Day-to-day variability in acceleration and MVPA (hereafter referred to as “variability”) was calculated for each participant using coefficient of variation (calculated as [standard deviation/mean of acceleration or MVPA across the seven measurement days] * 100%). In this study, coefficient of variation is an indicator of the variability in acceleration or MVPA and is a proxy for regularity of levels of acceleration or MVPA day-to-day (i.e., a lower coefficient of variation percentage indicates lower variability and therefore high regularity of day-to-day acceleration or MVPA).

### Acceleration/MVPA variability profiles

To further investigate variability, participants were separately grouped into tertiles of acceleration/MVPA (low; mid; high) and into tertiles of coefficient of variation (variability) (low; mid; high). Combinations of the groupings for acceleration/MVPA and variability were used to categorise participants into one of the following nine profiles: low acceleration/ MVPA and high/mid/low variability; mid acceleration/MVPA and low/mid/high variability; high acceleration/MVPA and low/mid/high variability.

### Statistical Analysis

All statistical analyses were conducted using STATA 16.1 (Stata Statistical Software: Release 16. College Station, TX: StataCorp LLC), with level of significance set at *p* < 0.05. All accelerometer data were visually checked for normality.

Descriptive statistics of the analytical sample (i.e., gender, age, occupation, education, and income) were summarised using percentages and frequencies. Characteristics of those who were included/excluded from the analysis sample were compared.

Individual values for acceleration and MVPA were calculated for each day of the week (i.e., Monday, Tuesday etc.) and used to derive group level data for each day of the week (i.e., means, SDs, ranges, medians, 25th and 75th percentiles, and outliers), which were presented using box-and- whisker plots.

Individual acceleration and MVPA data were used to estimate variability in these measures across the seven-day measurement period. For each participant, acceleration and MVPA were summed over the seven-day period and divided by seven to derive mean (SD) values, for calculation of variability in acceleration and MVPA. Estimates of variability were calculated for the whole sample, and for participants in each sociodemographic category, as shown in Table 2. Sociodemographic differences were assessed using t-tests (gender only) or one way analysis of variance (for age, occupation, education, and income). Individual combinations of acceleration/MVPA and their corresponding variability were plotted on scatter graphs to illustrate the nine profiles of these combined measures. Sociodemographic variables of participants in each of the nine profiles were summarised and displayed in a horizontal bar chart.

## Results

Of the 733 participants who provided accelerometer data, 612 participants (83%) provided valid accelerometer data for the seven days (i.e., 480 + minutes of valid wear time for measurement each day), providing a total of 4,284 days of observations. Sociodemographic variables are shown in Table 1. The average age was 60.6 (SD 6.9; range 48-73) years; there were more women than men, most had a university education and were either in professional jobs or not in the work force/retired. Younger adults and those who did not provide a valid answer to the education question were slightly more likely to be excluded from the analysis sample (see Supplementary Table 1).

**Table 1:** Sociodemographic variables of the participants. Brisbane, Australia 2014

**Characteristics**	**Analytical sample (*N* = 612)**	
	**N**	**%**
Gender		
Women	360	58.8
Men	252	41.2
Age (y)		
48–54	149	24.4
55–64	257	42.0
65 +	206	33.7
Occupation^a^		
Professionals	170	27.8
Blue collar	47	7.7
White collar	44	7.2
Office Workers	47	7.7
Not in the labour force/retired	216	35.3
No answer	88	14.4
Education		
Year 12 or less	170	27.8
Diploma or certificate	166	27.1
Bachelor degree or higher	233	38.1
No answer	43	7.0
Income (AUD per year)^b^		
< $41,599	116	19.0
$41,600-$72,799	133	21.7
$72,800-$129,999	149	24.4
≥ $130,000	109	17.8
No answer	105	17.0

^a^Professionals: professional and managers; Blue collar: technicians and trades workers, labourers and machinery operators and drivers; White collar: community and personal service workers and sales workers; Office workers: clerical and administrative workers; Not in the labour force: permanently unable to work, student and retired

^b^Australian dollar; annual household income

**Fig. 1:**
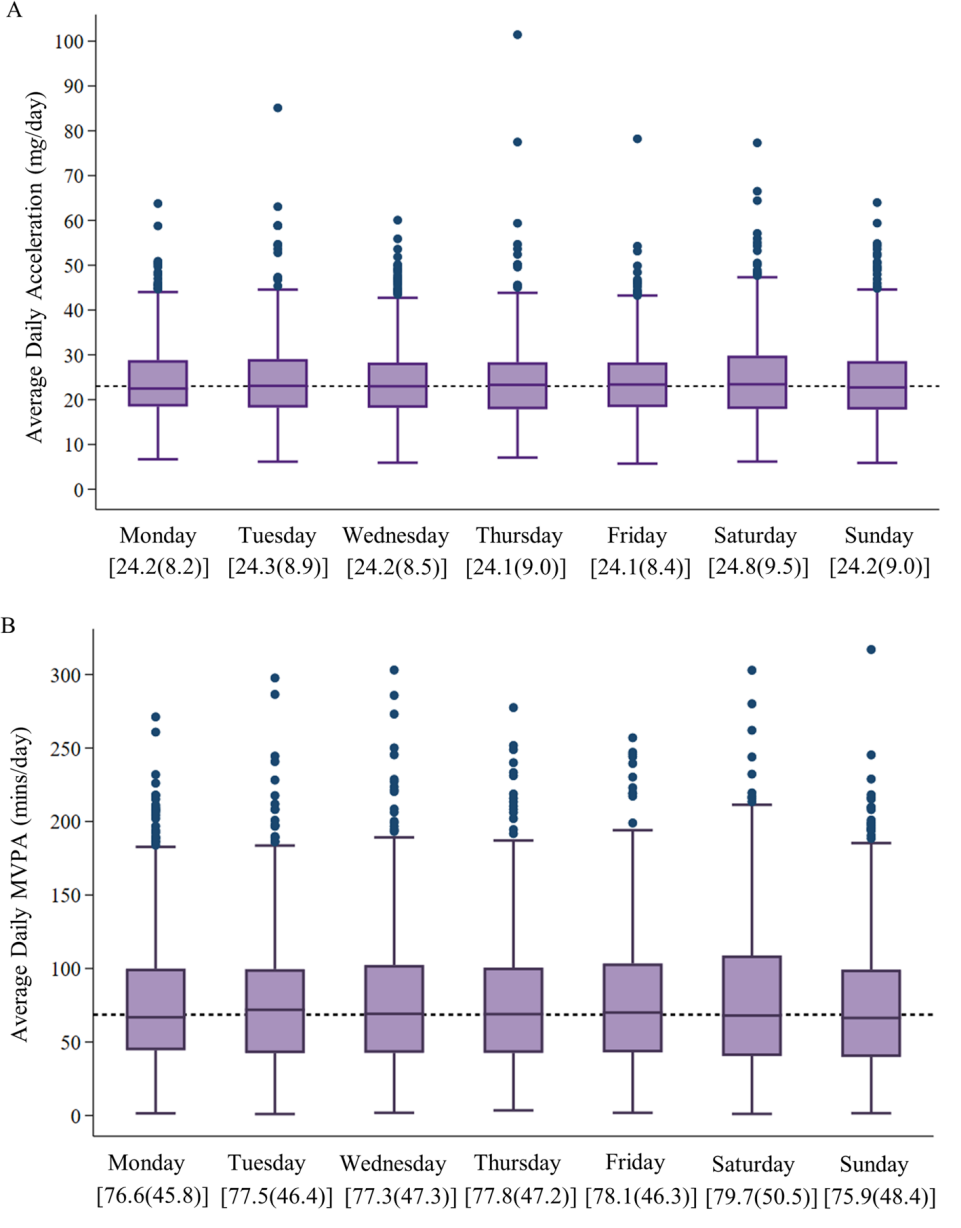
Box-and-whisker plots of the distributions of for each for (**A**) acceleration, and (**B**) MVPA. Brisbane, Australia 2014 (*N* = 612). Horizontal dotted line indicates daily acceleration (median: 23.5 m*g*/day) and MVPA (median: 70.6 mins/day). Values in parentheses represent means and standard deviation for each day for (**A**) acceleration and (**B**) MVPA. MVPA, Moderate-to-vigorous physical activity; m*g*, millgravity

Box and whisker plots of acceleration and MVPA are shown in Fig. 1A and B respectively for each day of the week. Mean acceleration was remarkably consistent across the week, ranging from 24.1 m*g*/day on Thursday and Friday to 24.8 m*g*/day on Saturday (see Fig. 1A). Differences in MVPA across days of the week were slightly more noticeable, mean values ranged from 75.9 mins/day on Sunday to 79.7 mins/day on Saturday (see Fig. 1B). There were however no significant differences in either acceleration or MVPA across days of the week when these group level data were considered.

Descriptions of acceleration and it’s variability, for the total sample, and according to sociodemographic variables are shown in Table 2. Over seven days, average acceleration was 24.3 (SD 7.2; range 7.3-65.8) m*g*/day, with average individual variability of 18.8% (SD 9.3%; range 3.4-87.7%). Average MVPA was 77.6 (SD 39.0; range 2.0-264.9) mins/day over the seven days, with average individual variability of 35.4% (SD 15.6%; range 7.3-124.6%).

There were significant differences in average daily acceleration across categories of age, occupation, and income (but not gender or education, see Table 2). The highest accelerations were observed in participants aged 48-54 years and in blue-collar workers. Men, participants aged 48-54 years, professionals, and those in the highest income category had high day-to-day variability in acceleration. However, among blue collar workers, who had the highest average daily acceleration (28.6 m*g*/day), variability (18.3%) was close to that of the group average (18.8%).

Time spent in MVPA also varied by age, occupation, and income, but not by gender and education. The highest durations of daily MVPA were observed in participants aged 48–54 years and in blue collar workers. Variability of MVPA was higher in men than women, but did not differ significantly across categories of the other sociodemographic indicators (Table 2). The most active groups (48-54 years and blue collar workers) had lower than average variability in MVPA.

**Table 2:** Description of average daily acceleration, average daily MVPA and their corresponding variability, according to sociodemographic variables. Brisbane, Australia 2014 (*N* = 612)

	**Acceleration (m*g*/day)**				**MVPA (mins/day)**			
	Mean (SD)^a^	*P* value^b^	Variability^c^ % (SD)	*P* value^b^	Mean (SD)^a^	*P* value^b^	Variability^c^ %(SD)	*P* value^b^
Total sample	24.3 (7.2)		18.8 (9.3)		77.6 (39.0)		35.4 (15.6)	
Gender		0.554		< 0.001		0.474		< 0.001
Women	24.4 (7.0)		17.3 (7.2)		76.6 (38.5)		32.7 (13.7)	
Men	24.1 (7.5)		21.0 (11.2)		78.9 (39.6)		39.1 (17.3)	
Age (y)		< 0.001		< 0.001		< 0.001		0.147
48–54	27.4 (7.7)		20.8 (11.3)		95.2 (40.1)		33.2 (14.9)	
55–64	24.3 (7.0)		19.1 (9.1)		78.4 (37.6)		36.0 (16.2)	
65 +	22.0 (6.2)		17.0 (7.4)		63.8 (34.3)		36.1 (15.3)	
Occupation^d^		< 0.001		< 0.001		< 0.001		0.075
Professionals	25.2 (7.6)		21.3 (11.0)		82.0 (36.7)		35.7 (16.2)	
Blue collar	28.6 (7.7)		18.3 (6.5)		102.5 (45.2)		31.9 (10.8)	
White collar	25.9 (7.0)		15.9 (6.0)		85.3 (42.3)		32.3 (13.0)	
Office Workers	24.4 (6.6)		17.6 (7.1)		80.8 (35.1)		31.8 (11.7)	
Not in the labour force/retired	22.3 (6.4)		17.4 (7.0)		67.2 (36.2)		36.0 (15.9)	
No answer	23.9 (7.2)		20.0 (12.5)		75.6 (38.8)		38.3 (18.3)	
Education		0.665		0.007		0.837		0.627
Year 12 or less	23.9 (7.1)		17.0 (7.0)		75.4 (39.5)		35.3 (14.2)	
Diploma or certificate	24.7 (7.8)		18.1 (8.4)		78.3 (39.3)		34.4 (14.8)	
Bachelor degree or higher	24.1 (7.0)		20.1 (10.3)		78.1 (38.6)		35.6 (16.6)	
No answer	25.0 (7.0)		20.7 (12.9)		80.5 (38.7)		37.8 (18.5)	
Income (AUD per year)^e^		< 0.001		< 0.001		< 0.001		0.298
< $41,599	21.8 (6.8)		16.3 (7.7)		64.4 (37.4)		35.4 (16.7)	
$41,600-$72,799	23.2 (6.5)		18.3 (7.8)		73.1 (38.8)		35.9 (16.2)	
$72,800-$129,999	25.9 (7.8)		18.4 (7.8)		87.0 (42.5)		33.0 (13.4)	
≥ $130,000	25.4 (6.9)		22.3 (10.9)		81.6 (30.6)		36.7 (15.2)	
No answer	24.9 (7.2)		19.1 (11.5)		80.2 (39.5)		36.6 (16.8)	

*SD* Standard Deviation, *Y* years, *mg* milligravity, *AUD* Australian Dollar, *MVPA* moderate-to-vigorous physical activity

^a^Average

^b^*P*-value

^c^Coefficient of Variation (%)

^d^Professionals: professional and managers; Blue collar: technicians and trades workers, labourers and machinery operators and drivers; White collar: community and personal service workers and sales workers; Office workers: clerical and administrative workers; Not in the labour force: permanently unable to work, student and retired

^e^Australian dollar; annual household income

**Fig. 2:**
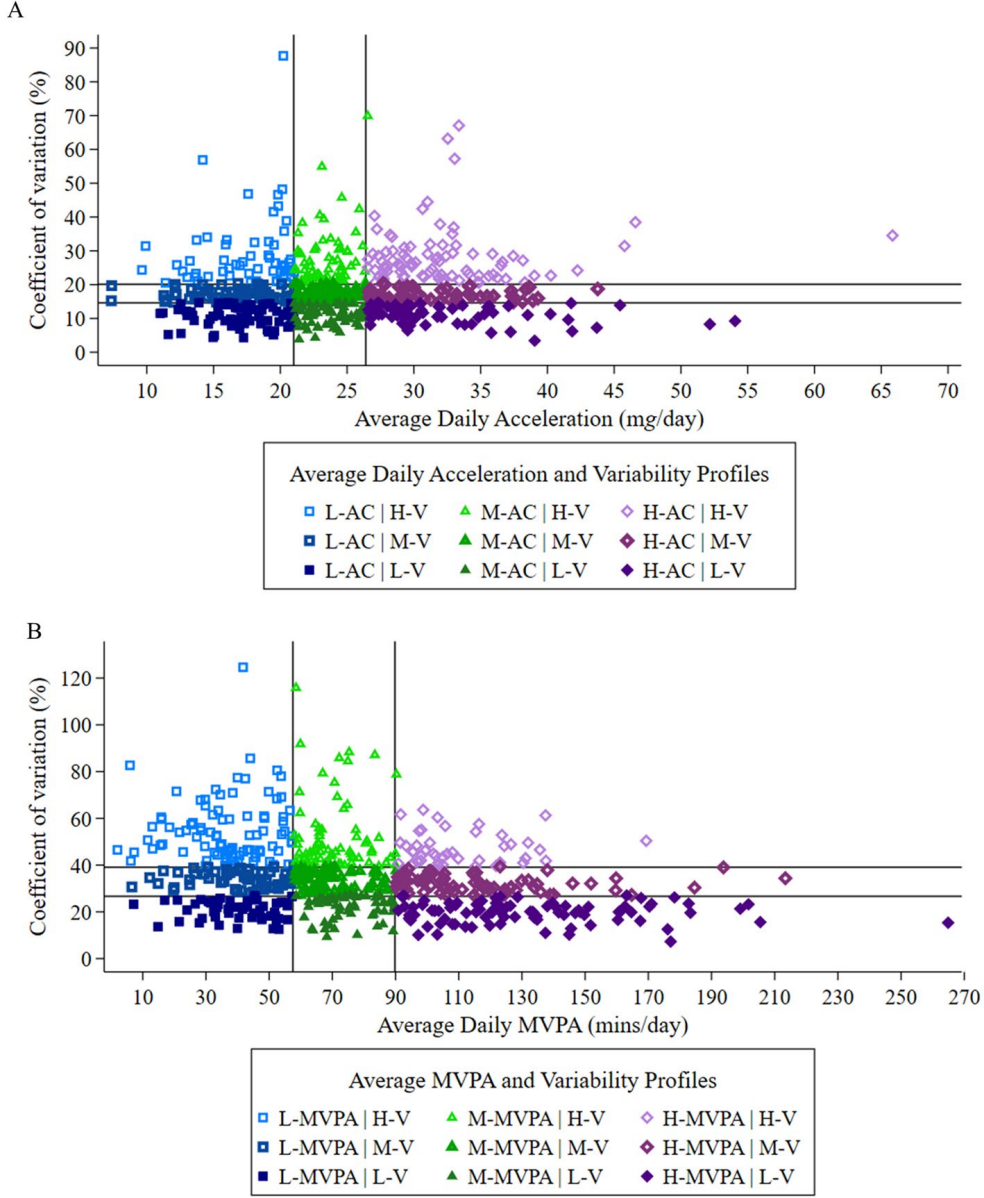
Scatter plot showing the distribution (with tertiles) of (**A**) average daily acceleration and it’s coefficient of variation and (**B**) of MVPA and it’s coefficient of variation. Brisbane, Australia 2014 (*N* = 612). Vertical lines represent 21.0 and 26.4 m*g*/day (**A**), 57.5 and 89.8 mins/day (**B**); horizontal lines represent 14.6% and 20.1% (**A**), 26.7% and 39.1% (**B**). L = Low; M = Mid; H = High; V = Variability; AC = Acceleration; MVPA = moderate-to-vigorous physical activity; m*g* = millgravity

Distribution of average daily acceleration and it’s variability, and of average daily MVPA and it’s variability, for individual participants in each of the nine variability profiles are shown in Fig. 2A and B respectively.

For acceleration, (Fig. 2A) over the seven days, participants in the low, mid, and high tertiles accumulated an average of 17.0 (SD 2.9; range 7.3-21.0), 23.6 (SD 1.5; range 21.1-26.5), and 32.2 (SD 5.3; range 26.6-65.8) m*g*/day respectively. Participants in the low, mid, and high variability categories had an average variability of 10.8% (SD 2.7%; range 3.4-14.7%), 17.1% (SD 1.6%; range 14.7-20.4%) and 28.5% (SD 9.4%; range 20.5-87.7%) respectively (Fig. 2A) (Acceleration and MVPA data for each of the nine groups for acceleration are shown in Supplementary Table 2).

For MVPA, (Fig. 2B) over the seven days, participants in the low, mid, and high categories accumulated an average of 38.8 (SD 12.9; range 2.0-57.8), 71.9 (SD 8.9; range 58.0-90.3) and 122.0 (SD 28.2; range 90.4-264.9) mins/day respectively. Participants in the low, mid, and high variability categories had an average variability of 20.5% (SD 4.5%; range 7.3-27.1%), 33.1% (SD 3.5%; range 27.1-39.4%) and 52.4% (SD 13.5%; range 39.4-124.6%) respectively (Acceleration and MVPA data for each of the nine groups for MVPA are shown in Supplementary Table 2).

**Fig. 3:**
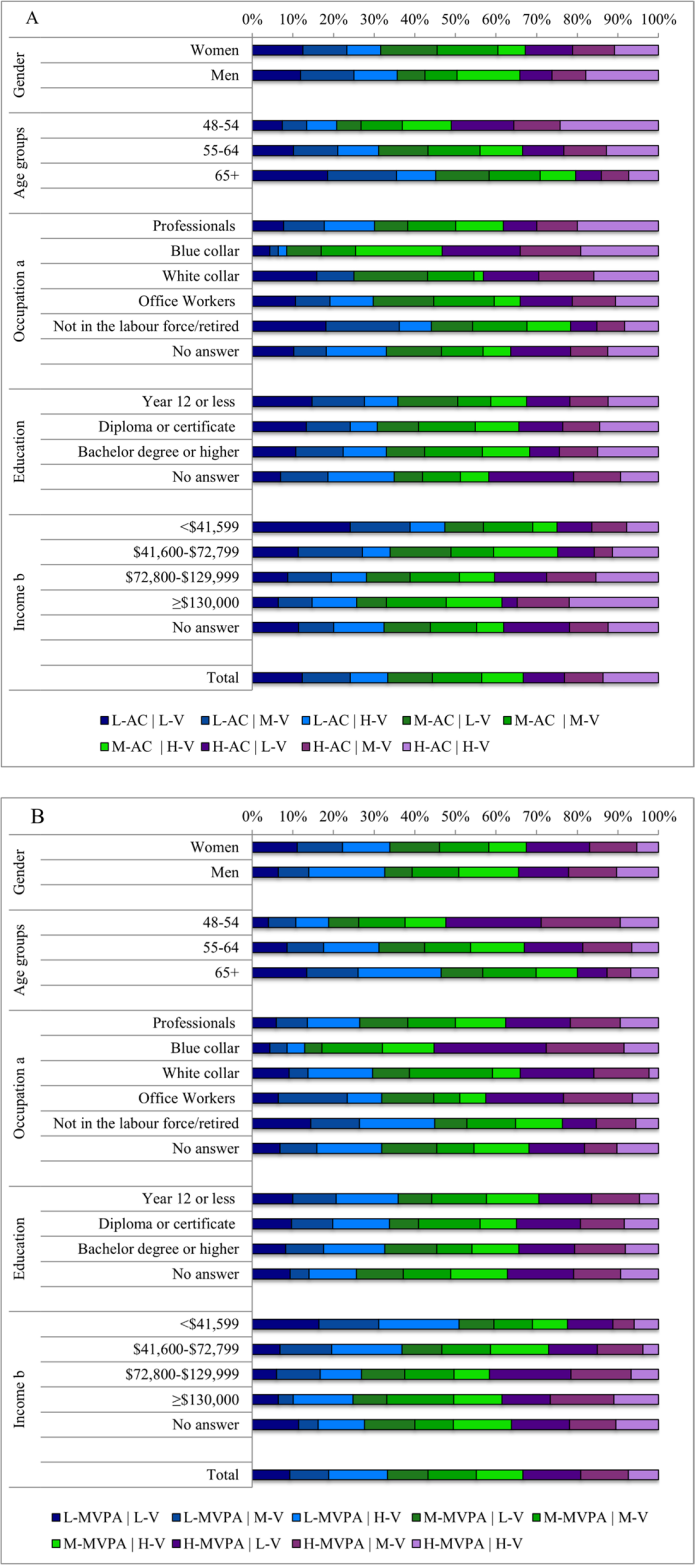
Proportion of acceleration (**A**), and MVPA (**B**) profiles based on acceleration or MVPA and their corresponding variability according to sociodemographic variables. Brisbane, Australia 2014 (*N* = 612). Bars from left to right represent low acceleration/ MVPA and low variability, low acceleration/MVPA and mid variability, low acceleration/MVPA and high variability, mid acceleration/MVPA and low variability, mid acceleration/MVPA and mid variability, mid acceleration/MVPA and high variability, high acceleration/MVPA and low variability, high acceleration/MVPA and mid variability and high acceleration/MVPA and high variability. ^a^Professionals: professional and managers; Blue collar: technicians and trades workers, labourers and machinery operators and drivers; White collar: community and personal service workers and sales workers; Office workers: clerical and administrative workers; Not in the labour force: permanently unable to work, student and retired. ^b^Australian dollar; annual household income. L = Low; M = Mid; H = High; V = Variability; AC = Acceleration; MVPA = Moderate-to-vigorous physical activity

Profiles of average daily acceleration and it’s variability and of MVPA and it’s variability are shown for participants in different sociodemographic categories in Fig. 3A and B respectively. Participants who were categorised as having high acceleration with high variability (14% of the total sample) were more likely to be 48-54 years, men, professionals or blue collar workers, and in the highest income category (than those in the other sociodemographic categories) (Fig. 3A). More participants were categorised in this profile than any other variability profile for acceleration. Participants who were categorised as having low acceleration with low variability (12% of the total sample) were most likely to be older (65 + years), retired or not in the work force, and in the lowest income category.

For MVPA, participants in the high MVPA and high variability profile group (7% of the total sample) were also more likely to be men and in the highest income category (than women and any other income group). In contrast, participants categorised as low MVPA and low variability (9% of the total sample) were more likely to be women, older (65 +) and not in the labour force/retired.

## Discussion

The aim of this study was to use accelerometer data to describe day-to-day variability in physical activity in a single week according to sociodemographic variables, in mid-aged Australian adults. The results show that group-level estimates of acceleration and MVPA, which are remarkably consistent across the week, conceal considerable individual differences in both overall amounts and variability of acceleration and MVPA. Average daily acceleration and MVPA varied according to age, occupation, income, but not gender and education, with the highest values observed in younger participants (48–54 years) and blue collar workers. Average variability in overall acceleration was quite low (18.8%), but differed across all sociodemographic characteristics, while variability in MVPA was greater (35.4%) and only differed by gender, and to a lesser extent by occupation.

In our study, variability in both acceleration and MVPA was lower in women than men, indicating that mid-age Australian women have more consistent day-to-day patterns of activity, or conversely, that physical activity varies more from day-day in mid-aged men. Although few previous studies have investigated day-to-day variability in activity, those that have also found lower variability in women [21]. A study which investigated day-to-day ‘patterns’ of work and leisure-time physical activity (in people age 18-65 in lower SES occupations) also found that men had more heterogenous activity patterns than women [22].

Both acceleration and MVPA varied across occupation groups, blue-collar workers (including technicians, trades workers, labourers, machinery operators and drivers) had the highest levels of activity, but their variability data indicate fairly consistent day-to-day patterns. Post-hoc investigation found that this group had higher activity on weekdays and lower activity on weekends, which is opposite to the pattern described in previous ‘weekend warrior’ research [23, 24]. The highest variability was seen in the professional and retired occupation groups, while white collar and office workers had low variability, indicting more day-to-day consistency in amounts of physical activity in the latter groups.

In this study, we used coefficient of variation to estimate variability. We selected this measure as it is useful for comparing two measures with different units and is easy to interpret, with higher values indicating greater day-to-day fluctuation in acceleration or MVPA, and lower values indicating more stability in daily physical activity across the week. Participants in the top tertiles of variability (> 20.5% for acceleration and > 39.4% for MVPA), as well as those in the bottom tertiles (< 14.7% for acceleration and < 27.1% for MVPA), were spread across a wide range of acceleration (< 21.0 to > 65.8 m*g*/day) and MVPA (< 57.8 to > 264.9 mins/day), indicating little association between overall volumes of activity and variability.

When we categorised participants into nine different profiles using tertiles of coefficient of variation and tertiles of acceleration or MVPA, we found that participants in every sociodemographic group were included in every activity profile, indicating wide heterogeneity in the how individuals in each group (e.g., blue or white collar workers) accumulate their daily activity. However, post-hoc investigation of individual accelerometer records found that many participants in the top tertiles of variability typically had one or two days where activity was different (markedly higher or lower) than on the other five or six days. This could indicate a 'weekend warrior' pattern (leisure time activity on the weekend), or high levels of activity at work, and lower levels on non-workdays. In general, participants with high variability *and* high activity were generally more socioeconomically advantaged (e.g., in professional occupations) while those with low activity and low variability tended to be women and older adults. As no previous studies have investigated variability in this way, there are no data from other studies for comparison. It would be interesting to investigate the multitude ways that the same overall weekly volume of acceleration/MVPA is accumulated by different individuals. Understanding how individuals accumulate their physical activity may be important in terms of development of physical activity promotion strategies for people with contrasting activity accumulation patterns. For example, among low active people, adding 5 mins a day might be a good target for people who currently have low daily variability (i.e., adding 5 mins a day to existing 10 mins per day), as this would continue their ‘low variability’ pattern. In contrast, adding 30 mins in one session per week might be preferred by people with a tendency for more variable physical activity patterns. Evidence suggest that mid-age adults are more likely to change their physical activity behaviour change if the intervention fits with their usual habits and preferences [21], and that smaller changes are easier to initiate and maintain than larger changes [25, 26].

## Strengths and Limitations

One strength of this study is that we measured acceleration and MVPA using accelerometers (which are less prone to social desirability or recall bias than subjective recall methods [27]), and provided novel data using ENMO and MVPA, as two different physical activity metrics. We also report raw accelerometer data, which allows comparability with data from different makes/models of accelerometers, and removes some of the researcher driven decisions on data processing, though these were still important for the MVPA estimates. However, as acceleration and MVPA are highly correlated, researchers should use caution when directly comparing these two metrics. Another strength is that the HABITAT participants were randomly selected from areas in Brisbane which span ten deciles of socio-economic status, which ensured inclusion of participants with a wide range of social and economic backgrounds [14, 28]. However, these data were from a sub-sample of the HABITAT cohort, which is a limitation, as the results may be less generalisable to all mid-age adults in Brisbane [15]. A second limitation is that exclusion of those who did not wear the accelerometer for the required number of hours and days, resulted in under inclusion of data from older participants, so our estimates of MVPA may be higher than in the general population of mid-age adults. A third limitation is each participant provided a single week of data, and variability estimates may be different at different times of the year/in different seasons [29, 30]. We also acknowledge that, because the accelerometers were not waterproof, we did not collect data during water-based activities, which may mean that estimates of acceleration, MVPA and/or variability were underestimated. However, in the baseline survey, only 10.7% of participants in the HABITAT cohort reported swimming once or more per week [17]. Finally, we acknowledge that using ENMO may be more difficult to interpret than time spent in any intensity category, and this metric cannot currently be compared with public health guidelines for physical activity.

## Conclusions

Our results show that group-level estimates of average acceleration and MVPA in a single week conceal considerable day-to-day variation in how mid-age Australians accumulate their acceleration and MVPA on a daily basis. There was no clear relationship between overall volume of activity and variability. Men, those in professional occupations and those not in the work force (retired) showed the greatest variability in MVPA, and women, older people, and those not in the labour force showed the least. Our study improves understanding of how mid-age individuals accumulate their physical activity beyond a sum of weekly minutes, but studies with larger sample sizes and longitudinal data are needed to increase the generalisability of these findings to other population groups. Future research should continue to use novel analysis techniques to describe patterns of acceleration and physical activity, and relationships between these patterns and health outcomes.

### Supplementary Information

**Additional file 1: Supplementary Table 1.** Sociodemographic variables of all participants in the HABITAT sub-study. Brisbane, Australia 2014.

**Additional file 2: Supplementary Table 2.** Mean and variability of acceleration (m*g*) and MVPA in each of nine categories based on tertiles of acceleration and its variability, and nine categories based on tertiles of MVPA and its variability. Brisbane, Australia 2014 (*N*=612).

## Data Availability

The datasets generated and/or analysed during the current study are not publicly, but de-identified data are available from the corresponding author on reasonable request.
