# Supplementary material for: Day-to-day variability in accelerometer-measured physical activity in mid-aged Australian adults
Source: BMC Public Health. 2023 Sep 28;23:1880. doi: 10.1186/s12889-023-16734-0 (PMC10540459; doi:10.1186/s12889-023-16734-0)
Supplement: Supplementary file 1 — Additional file 1: Supplementary Table 1. Sociodemographic variables of all participants in the HABITAT sub-study. Brisbane, Australia 2014. [file 12889_2023_16734_MOESM1_ESM.docx]

**Supplementary Table 1**: Sociodemographic variables of all participants in the HABITAT sub-study. Brisbane, Australia 2014.

| **Characteristics** | **Full sample (N= 733)** | | |
| --- | --- | --- | --- |
|  | **N** | **%** | **P Value ^b^** |
| Gender |  |  | 0.2 |
| Women  Men | 438  295 | 59.8  40.3 |  |
| Age (y) |  |  | 0.02 |
| 48-54  55-64  65+ | 191  307  235 | 26.1  41.9  32.1 |  |
| Occupation ^a^ |  |  | 0.07 |
| Professionals  Blue collar  White collar  Office Workers  Not in the labour force/retired  No answer | 214  58  51  54  244  112 | 29.2  7.9  7.0  7.4  33.3  15.3 |  |
| Education |  |  | <0.001 |
| Year 12 or less  Diploma or certificate  Bachelor degree or higher  No answer | 200  190  282  61 | 27.3  26.0  38.5  8.3 |  |
| Income (AUD per year) ^b^  <$41,599  $41,600-$72,799  $72,800-$129,999  ≥$130,000  No answer | 144  150  171  139  129 | 19.7  20.5  23.3  19.0  17.6 | 0.08 |

^a^ P-value

^b^ Professionals: professional and managers; Blue collar: technicians and trades workers, labourers and machinery operators and drivers; White collar: community and personal service workers and sales workers; Office workers: clerical and administrative workers; Not in the labour force: permanently unable to work, student and retired.

^c^ Australian dollar; annual household income.
