# Supplementary material for: Day-to-day variability in accelerometer-measured physical activity in mid-aged Australian adults
Source: BMC Public Health. 2023 Sep 28;23:1880. doi: 10.1186/s12889-023-16734-0 (PMC10540459; doi:10.1186/s12889-023-16734-0)
Supplement: Supplementary file 2 — Additional file 2: Supplementary Table 2. Mean and variability of acceleration (mg) and MVPA in each of nine categories based on tertiles of acceleration and its variability, and nine categories based on tertiles of MVPA and its variability. Brisbane, Australia 2014 (N=612). [file 12889_2023_16734_MOESM2_ESM.docx]

**Supplementary Table 2:** Mean and variability of acceleration (m*g*) and MVPA in each of nine categories based on tertiles of acceleration and its variability, and nine categories based on tertiles of MVPA and its variability. Brisbane, Australia 2014 (N=612)

|  |  | **Acceleration (m*g*/day)** | |  | **MVPA (mins/day)** | |
| --- | --- | --- | --- | --- | --- | --- |
| Variability profiles | **N** | Mean (SD)^a^ | Variability^b^ % (SD) |  | Mean (SD)^a^ | Variability^b^ % (SD) |
| Based on Acceleration | | | | | | |
| L-AC \| L-V | 75 | 17.2 (2.6) | 10.6 (2.9) |  | 18.8 (3.3) | 28.6 (10.7) |
| L-AC \| M-V | 72 | 16.6 (3.1) | 17.1 (1.5) |  | 17.7 (3.6) | 40.7 (14.2) |
| L-AC \| H-V | 57 | 17.4 (3.1) | 29.3 (11.2) |  | 17.1 (4.3) | 55.8 (19.9) |
| M-AC \| L-V | 67 | 23.6 (1.6) | 10.8 (2.6) |  | 24.1 (2.9) | 25.2 (9.1) |
| M-AC \| M-V | 74 | 23.6 (1.5) | 17.3 (1.7) |  | 23.5 (3.7) | 33.8 (10.2) |
| M-AC \| H-V | 63 | 23.5 (1.5) | 28.0 (8.7) |  | 23.1 (3.0) | 46.0 (14.2) |
| H-AC \| L-V | 62 | 32.7 (6.0) | 11.1 (2.6) |  | 33.5 (7.2) | 22.2 (7.7) |
| H-AC \| M-V | 58 | 31.7 (4.1) | 16.8 (1.6) |  | 30.2 (4.7) | 27.8 (8.4) |
| H-AC \| H-V | 84 | 32.3 (5.6) | 28.3 (8.5) |  | 29.8 (3.1) | 39.4 (11.9) |
| Based on MVPA | | | | | | |
| L-MVPA \| L-V | 56 | 40.5 (15.2) | 11.7 (4.3) |  | 40.5 (11.7) | 20.8 (4.0) |
| L-MVPA \| M-V | 59 | 40.8 (17.5) | 14.8 (4.9) |  | 40.5 (12.7) | 34.2 (3.2) |
| L-MVPA \| H-V | 89 | 47.0 (18.2) | 21.4 (10.6) |  | 36.6 (13.6) | 55.1 (13.4) |
| M-MVPA \| L-V | 61 | 70.1 (18.7) | 13.2 (5.1) |  | 75.4 (8.7) | 21.6 (5.0) |
| M-MVPA \| M-V | 73 | 71.4 (16.3) | 17.4 (4.6) |  | 70.2 (8.4) | 33.3 (3.8) |
| M-MVPA \| H-V | 70 | 74.1 (15.1) | 28.1 (10.9) |  | 70.7 (8.9) | 52.5 (15.7) |
| H-MVPA \| L-V | 87 | 121.2 (36.1) | 14.7 (6.0) |  | 132.4 (31.9) | 19.6 (4.3) |
| H-MVPA \| M-V | 72 | 119.3 (31.2) | 20.3 (5.6) |  | 117.0 (24.9) | 32.2 (3.3) |
| H-MVPA \| H-V | 45 | 116.0 (29.9) | 28.8 (10.6) |  | 109.8 (16.6) | 47.1 (6.7) |

^a^ Standard deviation

^b^ coefficient of variation (%)

L= Low; M= Mid; H= High; V= Variability; AC= Acceleration; MVPA= moderate-to-vigorous physical activity; m*g*= millgravity;
